# Chemical composition, antioxidant properties, and antifungal activity of wild *Origanum elongatum* extracts against *Phytophthora infestans*

**DOI:** 10.3389/fpls.2024.1278538

**Published:** 2024-01-26

**Authors:** Amal Hari, Ghizlane Echchgadda, Fatima-Azzahra Darkaoui, Noamane Taarji, Nihad Sahri, Mansour Sobeh, Said Ezrari, Salah-Eddine Laasli, Meryem Benjelloun, Rachid Lahlali

**Affiliations:** ^1^ Department of Plant Protection, Phytopathology Unit, Ecole Nationale d’Agriculture de Meknes, Meknes, Morocco; ^2^ Laboratory of Functional Ecology and Environmental Engineering, Faculty of Sciences and Technology, Sidi Mohamed Ben Abdellah University, Fez, Morocco; ^3^ AgroBioSciences Program, College of Agriculture and Environmental Sciences, Mohammed VI Polytechnic University, Ben-Guerir, Morocco; ^4^ Physio-Chemical Laboratory of Inorganic and Organic Materials (LPCMIO), Materials Science Center (MSC), Ecole Normale Supérieure, Mohammed V University in Rabat, Rabat, Morocco; ^5^ Microbiology Unit, Laboratory of Bioresources, Biotechnology, Ethnopharmacology and Health, Faculty of Medicine and Pharmacy Oujda, University Mohammed Premier, Oujda, Morocco

**Keywords:** *Solanum tuberosum* L., *Phytophthora infestans*, *Origanum elongatum*, chemical composition, antifungal activity

## Abstract

**Introduction:**

*Phytophthora infestans*, the causative agent of late blight disease, has gained notoriety for its destructive potential, leading to substantial losses in potato yields. Although conventional systemic fungicides have been shown to be effective in controlling plant pathogens, growing environmental concerns have prompted the need for more integrated disease management approaches. Hence, in this study, the effectiveness of wild *Origanum elongatum* extracts as biopesticides was explored in controlling *P. infestans* and potentially mitigating its devastating impact *in planta*.

**Methods:**

The aerial parts of *O. elongatum* were subjected to sequential extraction using water, hexane, chloroform, and methanol. The obtained extracts were tested *in vitro* through the poisoned food procedure for their capacity to obstruct *P. infestans* growth and to defeat potato blight severity *in vivo*. The phyto-contents (total phenolic content (TPC) and total flavonoid content (TFC)), as well as the antioxidant activities, were spectrophotometrically determined in all extracts, and the phytoconstituents of the most active extract (methanolic extract) were profiled *via* high-performance liquid chromatography–photodiode array–tandem mass spectrometry (HPLC–PDA–MS/MS).

**Results:**

*In vitro*, the complete inhibition rate of the *P. infestans* was obtained using the methanolic extract at 5 mg/mL, followed by the hexane and chloroform extracts at 10 mg/mL. Interestingly, complete inhibition of the pathogen was achieved upon the application of the aqueous extract at 10 mg/mL. *In vivo*, the aqueous extract at 25 mg/mL reduced the *P. infestans* severity rate to 27.25%, while the methanolic extract at 20 mg/mL led to the lowest severity rate. Moreover, the hexane and chloroform extracts impaired the pathogen severity rate to 50% and 41% using 20 mg/mL, respectively. The TPC and TFC in the extracts were variable with high concentrations detected in the methanolic extract with 485.42 mg GAE/g and 58.24 mg QE/g, respectively. In addition, the methanolic extract showed the highest antioxidant activities, while the chloroform extract exhibited the lowest activity. Liquid chromatography (LC)–MS/MS analysis of the methanol extract revealed 56 components from diverse classes. These included organic acids, phenolic acids, flavonoids, tannins, and coumarins.

**Conclusion:**

These findings suggest that *O. elongatum* could be investigated as a potential source of antifungal compounds targeting different phytopathogens.

## 1 Introduction

Multiple species of the genus *Phytophthora* cause numerous plant diseases that have significant economic implications. These include damping off, cankers, foliar disfigurements, and crown and root deteriorations (Agrios, 2005; Najdabbasi et al., 2020; Wang et al., 2020). *Phytophthora* species have wide host ranges, mechanisms of action, and geographical distributions (Scott et al., 2019; Moura et al., 2022). Among the devastating ailments affecting solanaceous plants, such as potatoes, late blight disease caused by the oomycete pathogen *Phytophthora infestans* is of significant concern (Yuen, 2021) as it can rapidly obliterate entire crops within 7 to 10 days, resulting in reduced potato production and escalated fungicide usage (Najdabbasi et al., 2020).

The estimated annual loss due to late blight alone amounts to approximately 6.7 billion dollars worldwide (Wang et al., 2021), with a considerable portion attributed to fungicide application costs (Guenthner et al., 2001; Najdabbasi et al., 2020). Despite advancements in cultural practices and disease prediction models, the breeding of resistant plant and chemical fungicide applications remains a vital component of late blight management programs (Haesaert et al., 2015; Yuen, 2021). However, *P. infestans*, owing to its rapid regeneration rates, unique metabolic pathways, and evolutionary adaptability, has developed resistance to several commercially available fungicides (Qin et al., 2016; Mazumdar et al., 2021). Furthermore, the incidence of late blight is currently on the rise, necessitating increased fungicide quantities to effectively treat *P. infestans* infections (Belay et al., 2021).

The use of synthetic fungicides to control *P. infestans* raises increasing concerns about their negative impacts on public health and environmental sustainability (Wightwick et al., 2010; Lamichhane et al., 2018). As a result, changing the paradigm to address this plant disease differently is imperative. Equally, this is suggested to maintain the continuity of food security and safety considered as the biggest obstacle facing the agricultural sector. Therefore, it is crucial to enhance strategies that reduce reliance on synthetic fungicides (Lamichhane et al., 2016).

Several studies have indicated that herbal-based natural combinations, such as plant extracts and oils (both vegetative and essential), exhibit significant efficacy against various plant pathogens (Soylu et al., 2006; Bajpai et al., 2009; Parveen et al., 2014; Dewitte et al., 2019). The effectiveness of medicinal plants is based on the abundance of their biologically active secondary metabolites. These phytochemicals possess potent antifungal properties that have been proven effective in disrupting microbial cell wall and membrane composition, altering hyphal morphology, inhibiting quorum sensing, impeding biofilm formation, and reducing mycotoxin production (Chen et al., 2013; Haque et al., 2016).

*Origanum elongatum* is a resilient plant species belonging to the *Origanum* genus, Lamiaceae family (Abdelaali et al., 2021). This endemic medicinal herb is predominantly found in Morocco (Figueredo et al., 2006), particularly in the northeastern region of the country, spanning from the Middle Atlas to the Rif mountain ranges (Figueredo et al., 2006; Bakha et al., 2020). It is primarily documented in high-altitude areas, such as the Bouyablane, Taounate, and Tazekka mountains (Tagnaout et al., 2020; Abdelaali et al., 2021). *O. elongatum* was reported to contain various classes of bioactive compounds, including terpenoids, hydrocarbon compounds, flavonoids, and phenolic compounds (Douhri, 2014; Oualili et al., 2019). The major volatile compounds present in this species are thymol, carvacrol, limonene, and linalool (Abdelaali et al., 2021).

The diverse array of chemical constituents in *O. elongatum* has demonstrated numerous biological activities such as hepatoprotective (Douhri, 2014), corrosion inhibition (El Attari et al., 2019), vasodilation (Yousfi et al., 2020), antioxidant (Oualili et al., 2019), antiparasitic (Ramzi et al., 2017), antiviral, antibacterial (Moussaoui, 2013; El Harsal et al., 2018; Boukhira et al., 2020), and antifungal properties (Amakran et al., 2014; Boukhira et al., 2020). Notably, despite the effectiveness of the plant in inhibiting various microorganisms, no studies have explored its effects against phytopathogens belonging to the *Phytophthora* genus.

This study aimed to investigate the antifungal potential of the aqueous and organic extracts from *O. elongatum* aerial parts through both *in vitro* and *in vivo* experiments. Furthermore, we explored their antioxidant potential, determined their phenolic and flavonoid contents, and profiled the phytochemical composition of the most active extract.

## 2 Materials and methods

### 2.1 Pathogen cultures

The *P. infestans* strain that was isolated and identified in potato tubers showed typical signs of rotting. It was preserved in the fungal culture facility at the National School of Agriculture of Meknes. To maintain its ability to cause disease, the strain was inoculated into recently wounded tubers and then cultured on Potato Dextrose Agar (PDA) medium at a temperature of 25°C for 48 h. The pathogen cultures were subsequently grown for 1 week at 25°C on a PDA medium supplemented with streptomycin sulfate (100 g/L).

### 2.2 Plant materials

The aerial parts of *O. elongatum* were collected in July 2020 from the forest of Mernissa (34°40′48″N and 4°15′0″W) located in the Mountains of Rif, Taounate province, Morocco. They were thoroughly cleaned and allowed to air-dry for 1 week.

### 2.3 Preparation of the aqueous extract

The aerial parts of *O. elongatum* were thoroughly cleaned and allowed to air-dry for 1 week in order to obtain the aqueous extract. Further, the dried material was ground into a fine powder using an automated grinder, and 20 g was subjected to extraction in distilled water (200 mL) using an ultrasonic machine for 10 min. The obtained aqueous extract was filtered, centrifugated, evaporated using a rotavapor, and then lyophilized.

### 2.4 Preparation of the organic extracts

The organic extractions involved cold maceration techniques described by Saldaña et al. (2017). In our case, three organic solvents were used, namely, methanol, hexane, and chloroform. For each solvent, the plant powder (250 g) was repeatedly macerated in 500 mL of methanol for 3 days. The mixture was then filtrated and evaporated in a rotary at reduced pressure and 60°C to evaporate the solvent. Further, each extract was filtered before solvent evaporation in a rotary evaporator with reduced pressure and controlled temperatures (69°C for hexane and 60°C for chloroform).

### 2.5 *In vitro* antifungal activity of the aqueous and organic extracts on mycelial growth

The efficacy of organic and aqueous extracts obtained from *O. elongatum* was evaluated for their antifungal properties against *P. infestans* using the poisoned food technique (El Khetabi et al., 2020). Streptomycin sulfate (100 g/L) was added to the PDA medium. The aqueous extract was tested at various concentrations (1, 1.5, 2.5, 5, 10, 15, 25, and 50 mg/mL) to determine its ability to inhibit the growth of *P. infestans* mycelium. In contrast, the hexane, methanol, and chloroform extracts were tested at concentrations of 1, 1.5, 2.5, 5, 10, 15, and 20 mg/mL. For the experimental setup, a 5-mm mycelial plug with the mycelial surface facing downward was taken from the actively growing edge of a 7-day-old colony and placed in the center of a Petri dish under aseptic conditions. Petri dishes containing pathogens without any extracts served as controls. Each treatment was performed in quadruplicate, and the entire experiment was repeated twice over time. After 7 days of incubation at 25°C, the antifungal effect of each treatment was assessed in terms of the inhibition rate of the pathogen’s growth using the following formula:


Inhibition rate (%) = (C−T/C) ×100,

where C denotes the diameter of the mycelia colony observed on the control plates, and T represents the diameter of the colony observed on the media supplemented with the extract.

In addition, EC_50_ values were obtained in the *in vitro* extract test using the linear regression equation:


Y=mX+b,

where *Y* is the response variable (e.g., mycelial growth inhibition), *X* is the independent variable (concentration of the treatment), *m* is the slope of the line, and *b* is the y-intercept.

Therefore, the EC_50_ was calculated using the following formula:


$$EC_{50}=\frac{Y_{half}-b}{m\times X},$$

where *Y_half_* is halfway between the baseline and maximum response.

### 2.6 Measurement of total phenolic compounds

The determination of the total phenolic contents (TPCs) in the plant extracts was conducted according to the methodology described by Hmidani et al. (2021). Briefly, 500 µL of distilled water and a 1/10 dilution of the Folin–Ciocalteu reagent were combined with 100 µL of the extract. Subsequently, 400 µL of a sodium carbonate solution (7.5% w/v) was added. The resulting mixture was left at room temperature for 60 min, and the absorbance was measured at 765 nm. A calibration curve was constructed using gallic acid as a standard. The TPC was quantified as milligrams of gallic acid equivalent (GAE) per gram of dry weight (DW).

### 2.7 Measurement of total flavonoid content

The determination of total flavonoid content (TFC) was conducted according to the methodology outlined by Hmidani et al. (2021). Initially, a 1-mL portion of the plant extract was combined with 4 mL of distilled water. Subsequently, 0.3 mL of a 5% sodium nitrite solution and 0.3 mL of a 10% aluminum chloride solution were added to the mixture. The resulting solution was then incubated at room temperature in test tubes for 5 min, after which 2 mL of 1 M sodium hydroxide was added. Distilled water was added to achieve a final volume of 10 mL. The mixture was thoroughly mixed, and the absorbance was measured at 510 nm. The TFC was determined by calculating the quantity of quercetin equivalent (QE) per gram of DW using a standard curve generated from a quercetin solution. The TFC results were expressed in milligrams of quercetin equivalent per gram of dry weight (mg QE/g DW).

### 2.8 Measurement of extraction yield

The extraction yield was used as an indicator of the extraction efficiency and effects of the extraction conditions. In our case, the extraction yield was calculated using the following formula: EY = (weight of dry extract/weight of dry plant) × 100. After grinding, the dry biomass was weighed. Further, the extraction yield was calculated for each extract solvent, and the results were presented in percentages.

### 2.9 DPPH radical scavenging power

The antioxidant potential of the plant extracts was evaluated by measuring their ability to scavenge the DPPH radical, following the method developed by Blois, with slight modifications (Xu et al., 2009). Briefly, 100 µL of different concentrations (30–480 µg/mL) of each plant extract was added to 1.9 mL of methanolic DPPH at different doses (0.3 mM). After incubating the mixture in the dark at room temperature for 20 min, the absorbance was measured at 517 nm. The resulting data, obtained from plotting the scavenging capacity against sample concentration, were utilized to calculate the EC_50_ values (μg/mL) using the following equation:


$$DPPH\;scavenging\;effect\;\left(\%\right)\;=\;\left[\left(A_{0}–A_{ex}\right)/A_{0}\right]\;\times 100,$$

where *A*_0_ is the absorbance value of the control and *A_ex_* is the absorbance value in the presence of the extracts.

### 2.10 *In planta* antifungal effect of the plant extracts on late blight disease

Potato tubers from the cv. de Spunta variety, which is known to be sensitive to *P. infestans*, were utilized in the pot trials. The tubers were cleaned with tap water, disinfected for 5 min with a 10% solution of sodium hypochlorite, and then allowed to air-dry. Subsequently, the tubers were transferred to pots within a greenhouse environment, where a mixture of soil, peat, and sand in equal proportions (1:1:1) was used as the planting medium. The pots were regularly watered. The aqueous extract (10, 15, and 25 mg/mL) and the organic extracts (5, 10, and 20 mg/mL) were sprayed on both sides of the leaf surface of 10-week-old plants using an atomizer, along with distilled water as a negative control. *P. infestans* was sprayed on both control and treated plants at a dosage of 1 × 10^4^ sporangia/mL. Plants that had not received the plant extract treatments or been infected with the fungus were used as negative controls. The concentrations tested were chosen based on *in vitro* assays and, together with the three controls, were established in five blocks as part of the experiment. This trial was repeated twice over time.

The disease severity was assessed individually for each plant by estimating the leaf area affected by *P. infestans* on a percentage basis 10 days after the inoculation. The area under the disease progress curve (AUDPC) was assessed for each treatment by recording disease severity at 10-day intervals from the 1^st^ to the 40^th^ day after inoculation. AUDPC was calculated using the following equation:


$$\text{AUDPC }=\sum_{i=1}^{n-1}\frac{y_{i}+y_{i+1}}{2}\times \left(t_{i+1}-t_{i}\right),$$

where *y_i_* is the assessment of the disease severity at the *i*th observation, *t_i_* is the time in days at the *i*th observation, and *n* is the total number of observations.

### 2.11 HPLC–PDA–MS/MS

The annotation of the secondary metabolites of the plant’s methanolic extract was performed using high-performance liquid chromatography–photodiode array–tandem mass spectrometry (HPLC–PDA–MS/MS). A triple quadrupole spectrometer with an electrospray ionization (ESI) source was used in conjunction with a SHIMADZU LC-MS 8050 (Shimadzu, Kyoto, Japan) LC system. The separation procedure was carried out using a C18 reversed-phase column (Zorbax Eclipse XDB-C18, quick resolution, 4.6 × 150 mm, 3.5 µm Agilent, Santa Clara, CA, USA) with a flow rate of 1 mL/min. Gradients of water and acetonitrile (ACN) containing 0.1% formic acid each were used to go from 5% to 90% ACN over the course of 60 min. Using an autosampler (SIL-40C xs), 10 µL of the sample was automatically injected with a concentration of 10 mg/mL. The device was controlled by Shimadzu’s (Japan) LC solution software. The MS was operated in the negative ion mode. The *m*/*z* range used for the measured MS data was 100 *m*/*z* to 1,500 *m*/*z*, with two energy values of 35 eV and 45 eV.

### 2.12 Statistical analysis

All recorded variables were tested for normality using the Kolmogorov–Smirnov test. For the *in vitro* assays, the efficiencies of the antifungal activity were compared using the analysis of variance (ANOVA) procedure of the SPSS software (SPSS 20.0, SPSS Inc., Chicago, IL, USA). For the AUDPC, concentrations were compared using a t-test for all the extracts. The *in vitro* inhibition rate of mycelium growth for each treatment was transformed into ARCSIN (% inhibition) and presented as log_10_ values to meet normality assumptions. Mean values were compared using Tukey’s test at a significance level of *p* < 0.05. In order to highlight potential correlations between phenolic contents, the antioxidant capacity, and their biocontrol activity, a correlation analysis (Pearson’s type) was conducted at a significance level of 5%.

## 3 Results

### 3.1 *In vitro* antifungal activity of *O. elongatum* organic extracts

**Figure 1:**
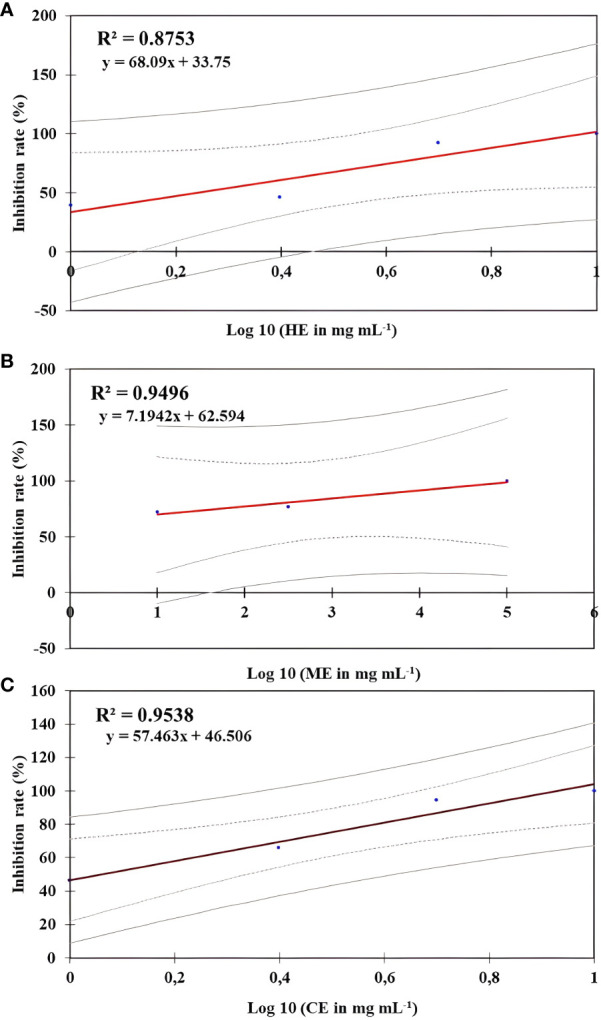
Regression curve effect of *Origanum elongatum*’s organic extracts on the mycelial growth of *Phytophthora infestans*. **(A)** Hexane extract (HE), **(B)** methanol extract (ME), and **(C)** chloroform extract (CE).

The *in vitro* effect of *O. elongatum* organic extracts on the mycelial growth of *P. infestans* is presented in **Table 1**. Noticeably, the three organic extracts exhibited a significant inhibitory effect (*p* < 0.05) on the mycelial growth of *P. infestans*. In the concentration range of 1 to 5 mg/mL, the inhibition rates were different among the tested extracts. The highest values of inhibition were obtained by the methanolic extract (72.18% to 100%), followed by the chloroform (46.32% to 100%) and hexane (39.2% and 100%) extracts. Further increase of the concentration to 10 mg/mL or 20 mg/mL resulted in a complete inhibition rate (100%). The linear regression equations were used to determine the estimated EC_50_ value, reflecting a 50% suppression of mycelial development. The findings are shown in **Figure 1**. The estimated EC_50_ for the methanolic was 0.01 mg/mL, followed by the chloroform (EC_50_ = 1.15 mg/mL) and hexane (1.73 mg/mL) extracts.

**Table 1:** *In vitro* effect of *Origanum elongatum* organic extracts on the growth of *Phytophthora infestans*.

Treatments (mg/mL)	CE	ME	HE	*p*
**1**	46.32 ± 0.43^b^	72.18 ± 1.46^a^	39.2 ± 1.569^c^	< 0.001
**2.5**	65.82 ± 1.31^b^	76.75 ± 1.46^a^	63.48 ± 2.72^c^	0.008
**5**	93.95 ± 0.99^a^	100 ± 0.00^a^	92.23 ± 1.16^a^	–
**10**	100 ± 0.00^a^	100 ± 0.00^a^	100 ± 0.00^a^	–
**20**	100 ± 0.00^a^	100 ± 0.00^a^	100 ± 0.00^a^	–
UC	0.00 ± 00			

The growth inhibition is expressed in percentage (%). Letters in superscript (a, b, and c) indicate the statistical difference at *p* < 0.05.

CE, chloroform extract; ME, methanolic extract; HE, hexane extract; UC, untreated control.

### 3.2 *In vitro* antifungal activity of *O. elongatum* aqueous extract

**Figure 2:**
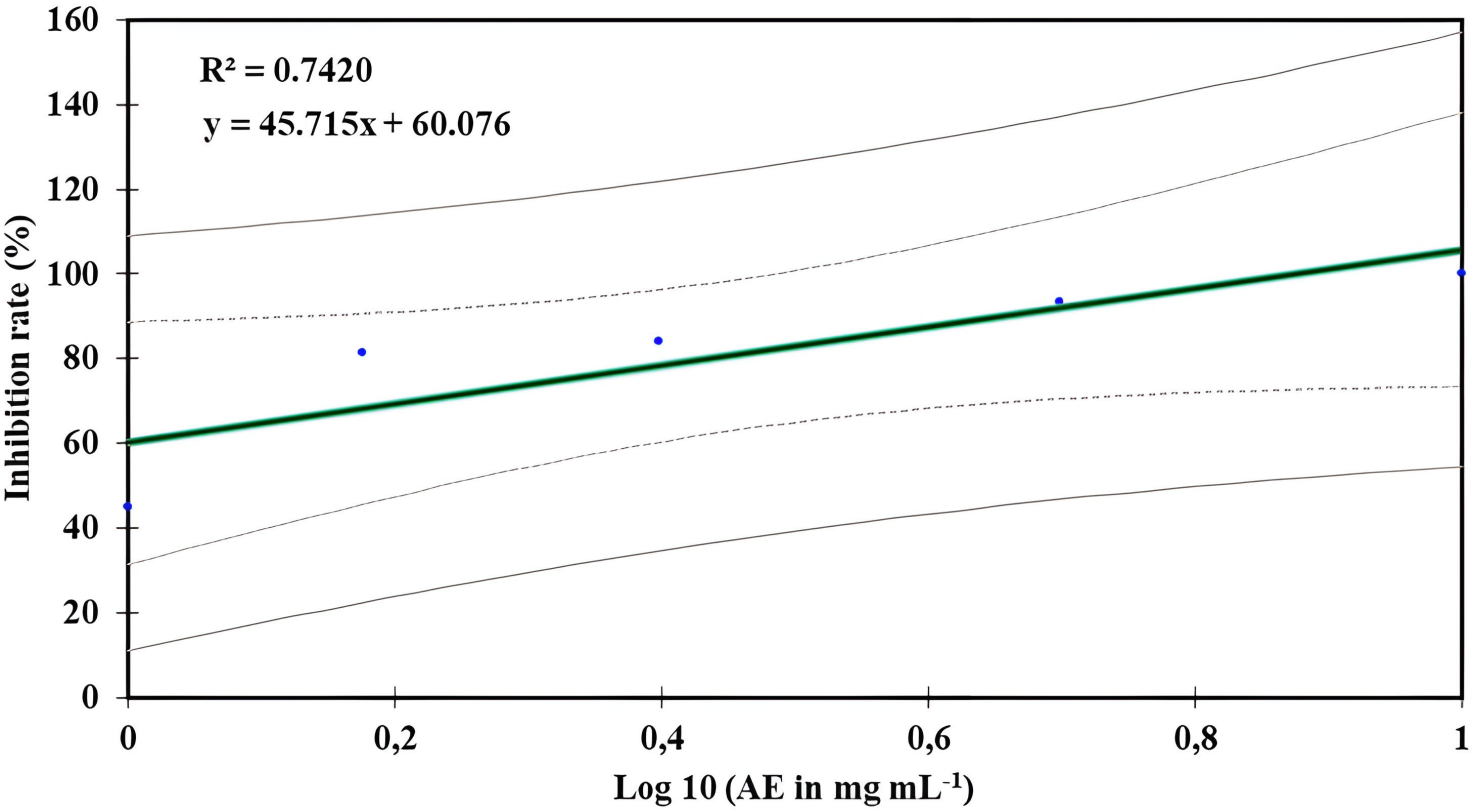
Regression curve effect of *Origanum elongatum*’s aqueous extract (AE) on the mycelial growth of *Phytophthora infestans*.

**Table 2** displays the *in vitro* activity of *O. elongatum* aqueous extract on the growth of *P. infestans*’ mycelium. The inhibition rate of *P. infestans* was positively correlated to the concentrations tested (*p* = 0.003) (**Figure 2**). The lowest inhibition rate was recorded at 1 mg/mL, while the highest inhibition rate of 100% was obtained at 10 mg/mL. In contrast, the correlation between the concentration of the aqueous extracts and the inhibition rate of *P. infestans* was tested with linear regression. As a result, the inhibition rate increased with the augmented concentration of the extract. Additionally, the EC_50_ value was established using linear regression models, and the results are presented in **Figure 2**. The estimated EC_50_ value was 0.60 mg/mL.

**Table 2:** *In vitro* effect of *Origanum elongatum* aqueous extract on the growth of *Phytophthora infestans*’ mycelium following 7 days of incubation.

Treatments (mg/mL)	Inhibition rates of *P. infestans* growth (%)
UC	0 ± 0.00^e^
1	45.14 ± 1.23^d^
1.5	81.34 ± 3.42^c^
2.5	84.18 ± 2.29^c^
5	93.62 ± 0.65^b^
10	100.00 ± 0.00^a^
15	100.00 ± 0.00^a^
25	100.00 ± 0.00^a^
50	100.00 ± 0.00^a^

The values are followed by standard errors (SE). Letters in superscript (a, b, and c) indicate the statistical difference at *p* < 0.05.

UC, untreated control.

### 3.3 *In planta* effect of *O. elongatum* organic and aqueous extracts

#### 3.3.1 Effect of *O. elongatum* organic and aqueous extracts on late blight disease

*In vivo*, *O. elongatum* extracts demonstrated noteworthy antifungal activity, exerting a significant inhibitory effect on infection severity (*p* ≤ 0.01). This effect was observed for both organic and aqueous extracts with different degrees depending on the type of extract and concentration (**Table 3**). The majority of the concentrations tested from both aqueous and organic extracts induced significantly lower disease severity index scores compared to the control group (100%). It is important to note that lower severity values indicate higher effectiveness of the extracts. Notably, the aqueous extract at a concentration of 25 mg/mL exhibited the most potent inhibitory effect against *P. infestans* (*p* < 0.001), while 10 mg/mL elicited the lowest inhibitory effect. Moreover, the efficacy of the methanol extract (ME) was found to be remarkable as it demonstrated the highest inhibition of late blight disease severity, reaching approximately 95% at a concentration of 20 mg/mL. Similar inhibitory effects were observed for all tested organic extracts with dose-dependent efficiencies. Both ME and chloroform extract (CE) displayed significantly lower severity rates compared to hexane extract (HE). At a concentration of 10 mg/mL, CE showed the highest severity, followed by ME, while the lowest severity was observed using HE. At a concentration of 20 mg/mL, ME exhibited the lowest severity, followed by EC and HE, respectively (**Table 3**).

**Table 3:** Effect of *Origanum elongatum* extracts on the disease severity *Phytophthora infestans* noted after 10 days of incubation at 25°C on potato leaves.

Extracts	C1	C2	C3	*p*
AE	69.5 ± 9.11^a^	60.25 ± 12.55^a^	27.25 ± 6.65^b^	<0.001
ME	74.25 ± 10.34^a^	64.25 ± 1.26^a^	5.75 ± 1.75^b^	<0.001
CE	75.25 ± 4.03^a^	66.50 ± 8.18^a^	41.00 ± 4.54^b^	<0.001
HE	68.75 ± 1.89^a^	57.25 ± 3.40^ab^	50.00 ± 1.41^b^	<0.001
UC	100 ± 0.00			

Concentrations of the aqueous extract: C1 = 10 mg/mL, C2 = 15 mg/mL, and C3 = 25 mg/mL. Concentrations of the organic extracts: C1 = 5 mg/mL, C2 = 10 mg/mL, and C3 = 20 mg/mL. The disease severity is expressed in percentage (%). The values are followed by standard errors (SE). Letters in superscript (a, b, and c) indicate the statistical difference at *p* < 0.05.

AE, aqueous extracts; CE, chloroform extract; ME, methanolic extract; HE, hexane extract; UC, untreated control.

#### 3.3.2 Effect of *O. elongatum* organic and aqueous extracts on *P. infestans* AUDPC

The values of AUDPC of both organic and aqueous extracts of *O. elongatum* are presented in **Table 4**. The lowest values of AUDPC indicate great effectiveness against *P. infestans*. The findings indicated dose-dependent inhibitory effects using aqueous and organic extracts (*p* < 0.001). In terms of comparison among organic extracts, HE showed the lowest inhibitory effects against *P. infestans* in all tested concentrations compared to CE and ME (*p* < 0.05). In 20 mg/mL and 10 mg/mL, the ME showed the highest inhibitory capacity, while in 5 mg/mL, the highest effectiveness was detected in CE (*p* < 0.05).

**Table 4:** Comparison of AUDPC values between the extracts and concentrations.

Extracts	C1	C2	C3	*p*
AE	2,194 ± 165.73^a^	1,021 ± 124.76^b^	927.5 ± 127.54^b^	0.0007
ME	1,880 ± 344.83^a^	350 ± 34.15^b^	258 ± 354.71^b^	0.0059
HE	2,475 ± 260.88^a^	2,098 ± 193.21^a^	2,214.8 ± 30.37^a^	0.0431
CE	1,913 ± 144.16^a^	1,288 ± 409.82^b^	929 ± 293.60^b^	0.0321

Concentrations of the aqueous extract: C1 = 10 mg/mL, C2 = 15 mg/mL, and C3 = 25 mg/mL. Concentrations of the organic extracts: C1 = 5 mg/mL, C2 = 10 mg/mL, and C3 = 20 mg/mL. The values are followed by standard errors (SE). Letters in superscript (a, b, and c) indicate the statistical difference at *p* < 0.05.

AE, aqueous extracts; CE, chloroform extract; ME, methanol extract; HE, hexane extract.

#### 3.3.3 Extraction yield and total phenolic and flavonoid contents

The extraction yield (%) was different among the tested extracts of *O. elongatum* (**Table 5**). The highest yield was recorded in the AE, followed by the ME and CE. The lowest extraction yield was recorded using hexane. The flavonoid contents in *O. elongatum* aerial parts were estimated as mg quercetin equivalents per gram of extract (mg QE/g). The TFC in different *O. elongatum* organic and aqueous extracts ranged between 12.52 ± 0.13 and 58.24 ± 0.15 mg/g of extract (**Table 5**). The HE presented the lowest TFC (12.52 ± 0.13 mg QE/g of extract), while the highest contents were detected in the ME (58.24 ± 0.15 mg QE/g of the extract). The TPC values were estimated as milligrams of GAE per gram of extract. TPC of *O. elongatum* organic and aqueous extracts ranged between 50.95 ± 0.15 mg and 485.42 ± 1.04 mg GAE/g (**Table 5**). The polyphenols in all extracts were significantly different (*p* < 0.001). The HE and ME contained the lowest (50.95 ± 0.15 mg GAE/g) and highest (485.42 ± 1.04 mg GAE/g) TPCs, respectively.

**Table 5:** Extraction yield (%), total flavonoid, and phenolic contents of the aqueous and organic extracts of *Origanum elongatum*.

Extracts	Extraction yield (%)	TPC	TFC
AE	37.45	83.22 ± 0.40^bc^	46.13 ± 0.10^a^
ME	34.42	485.42 ± 1.04^a^	58.24 ± 0.15^a^
CE	22.39	136.93 ± 0.22^b^	20.73 ± 0.48^b^
HE	9.72	50.953 ± 0.15^c^	12.52 ± 0.13^bc^

The TPC and TFC values are followed by standard errors (SE). Letters in superscript (a, b, and c) indicate the statistical difference at *p* < 0.05.

AE, aqueous extract; CE, chloroform extract; ME, methanolic extract; HE, hexane extract; TPC, total polyphenol content; TFC, total flavonoid content.

#### 3.3.4 DPPH radical scavenging

The results of the antioxidant effect of the aqueous and organic extracts of *O. elongatum* are displayed in **Table 6**. From the analysis of EC_50_ values, the DPPH radical scavenging capacities of all tested extracts (aqueous and organic) were remarkably different from each other (*p* < 0.05). The lowest EC_50_ value was recorded for ME, followed by HE and AE. The less active extract was the CE as evidenced by a high EC_50_ value (**Table 6**).

**Table 6:** *In vitro* antioxidant effect (EC_50_) of the aqueous and organic extracts *Origanum elongatum* using DPPH assay.

Extract	DPPH scavenging activity (EC_50_ (μg/mL)
ME	87.66 ± 0.57 ^cd^
HE	99.60 ± 0.52^c^
AE	133.25 ± 0.43^b^
CE	162.55 ± 0.43^a^

The values are followed by standard errors (SE). Letters in superscript (a, b, and c) indicate the statistical difference at *p* < 0.05.

AE, aqueous extract; CE, chloroform extract; ME, methanolic extract; HE, hexane extract.

The correlation between different phenolic contents and antioxidant capacities with biocontrol activities of *O. elongatum* revealed a negative relationship between inhibitory activity and TPC in both methanolic and chloroform extracts, while it was positive in hexane and aqueous extracts. Regarding TFC, all correlation trends with biocontrol activity were positive in all extracts. Likewise, positive correlations were observed between the antifungal effect and DPPH except in hexane extract (**Table 7**).

**Table 7:** Pearson’s correlation coefficient between antifungal inhibition and TPC, TFC, and DPPH of different plant extracts.

Extract (IR)	TPC	TFC	DPPH
ME	−0.961	0.735	0.943
HE	0.982	0.976	−0.543
CE	−0.689	0.579	0.993
AE	0.924	0.189	−0.520

AE, aqueous extract; CE, chloroform extract; ME, methanolic extract; HE, hexane extract; IR, inhibition rate.

#### 3.3.5 HPLC–PDA–MS/MS

**Figure 3:**
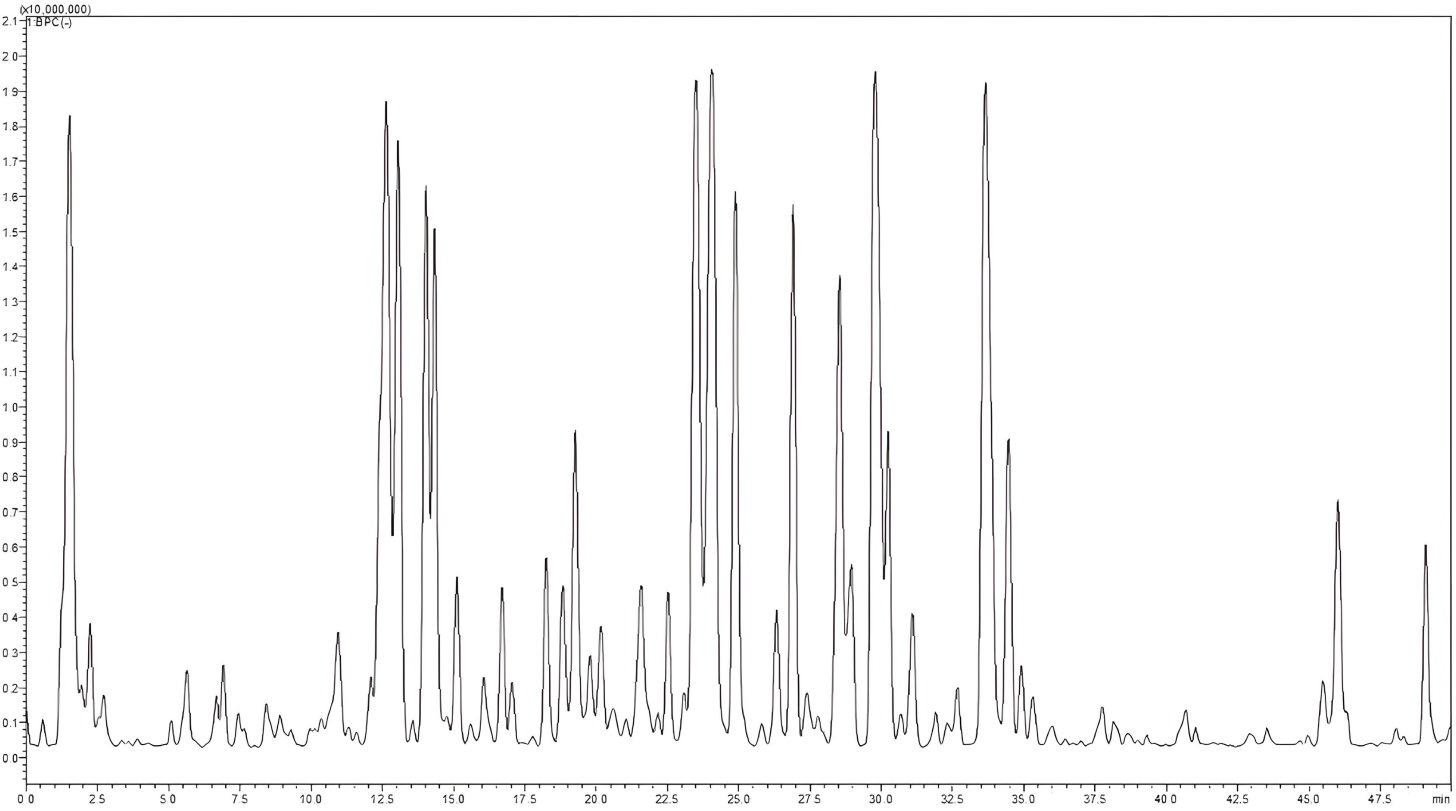
LC–MS profile of *Origanum elongatum* aerial parts methanolic extract. LC–MS, liquid chromatography–mass spectrometry.

A total of 56 components from diverse classes were tentatively characterized in the methanolic extract of *O. elongatum* (**Table 8**). These included organic acids, phenolic acids, flavonoids, tannins, and coumarins. Caffeic acid, rosmarinic acid, and their derivatives are examples of detected phenolic acids (**Figure 3**).

**Table 8:** Annotated compounds from *Origanum elongatum* extract using LC–MS/MS.

No.	Rt (min)	[M–H] -	MS/MS	Proposed compounds
1	0.91	191	111, 191	Citric acid
2	1.51	191	108	Quinic acid
3	1.61	133	115, 133	Malic acid
4	2.10	125	125	Pyrogallol
5	5.42	315	109, 153	Protocatechuic acid glucoside
6	5.54	167	109	Vanillic acid
7	5.88	329	167	Vanillic acid glucoside
8	6.45	153	109, 153	Dihydroxybenzoic acid
9	7.82	503	161, 179	Caffeic acid diglucoside
10	8.73	339	133, 177	Esculetin glucoside
11	8.87	137	109, 137	Hydroxybenzoic acid
12	9.03	299	137	Hydroxybenzoic acid glucoside
13	9.28	313	135, 151	Vanillin glucoside
14	9.85	475	133, 177	Esculetin galloyl rhamnoside
15	10.45	341	135, 179	Caffeic acid glucoside
16	10.53	325	119, 163	Coumaric acid glucoside
17	11.19	353	135, 179, 191	Chlorogenic acid
18	12.01	179	135, 179	Caffeic acid
19	12.21	305	151, 225	(epi)Gallocatechin
20	12.29	163	119	Coumaric acid
21	13.93	359	197	Syringic acid glucoside
22	14.22	593	353, 383	Apigenin C-diglucoside
23	16.04	637	285	Kaempferol sinapoyl rhamnoside
24	17.43	623	285	Kaempferol glucoside glucuronide
25	18.26	339	163	Coumaric acid glucuronide
26	18.36	537	179, 197	Salvianolic acid J
27	18.99	431	269	Apigenin glucoside
28	19.61	463	271, 301	Quercetin glucoside
29	19.69	177	133, 177	Esculetin
30	19.92	579	285, 447	Kaempferol glucoside arabinoside
31	20.06	447	285	Kaempferol glucoside
32	20.14	461	285	Kaempferol glucuronide
33	21.00	555	135, 161, 197	Salvianolic acid K
34	21.21	521	161, 179, 197	Syringic acid caffeoyl glucoside
35	21.53	447	285	Kaempferol glucoside
36	22.09	515	179, 191	Dicaffeoylquinic acid
37	23.09	477	271, 301	Quercetin glucuronide
38	23.71	515	353	Dicaffeoylquinic acid
39	24.02	359	135, 161, 197	Rosmarinic acid
40	24.10	579	285	Kaempferol pentosyl glucoside
41	24.18	417	285	Kaempferol rhamnoside
42	24.29	555	359	Salvianolic acid K isomer
43	24.98	549	161, 387	Caffeoyl ethyl rosmarinate
44	25.72	621	285	Kaempferol quinyl glucoside
45	26.17	537	179, 197	Melitric acid A
46	26.77	717	321, 519	Salvianolic acid B
47	26.82	519	293, 321	Melitric acid B
48	27.03	609	179, 285	Kaempferol caffeoyl glucoside
49	29.35	493	161, 179	Salvianolic acid A
50	30.91	607	284, 299	Diosmetin caffeoyl glucoside
51	33.63	271	151	Naringenin
52	34.31	269	151, 269	Apigenin
53	34.91	301	301	Quercetin
54	37.84	551	193, 359	Schizotenuin F
55	46.03	285	151, 271, 285	Kaempferol
56	49.10	327	241, 269	Apigenin acetate

LC–MS/MS, liquid chromatography–tandem mass spectrometry.

## 4 Discussion

Plant-based biopesticides have gained significant attention in agriculture due to their capacity to protect crops from pathogens, pests, and weeds. Indeed, phytoconstituents were extensively demonstrated to exhibit antimicrobial, insecticidal, herbicidal, larvicidal, allelopathic, repellent, and nematicidal properties (Kisiriko et al., 2021; Bitchagno et al., 2022). This work aimed to explore the potential of *O. elongatum* extracts as biocontrol agents against *P. infestans*, the pathogen responsible for late blight disease in potato crops. Notably, this study represents the first instance of demonstrating the antifungal properties of both aqueous and organic (chloroform, methanol, and hexane) extracts derived from *O. elongatum* against *P. infestans* using *in vitro* and *in vivo* approaches. Furthermore, the total polyphenol and flavonoid contents, as well as the antioxidant capacity of the extracts, were determined. Additionally, the methanolic extract was subjected to phytochemical annotation to identify the composition of its secondary metabolites using HPLC–PDA–MS/MS analysis.

The TFC in organic and aqueous extracts of *O. elongatum* showed significant variations. The hexane extract had the lowest TFC (12.52 ± 0.13 mg QE/g of extract), while the methanolic extract had the highest levels (58.24 ± 0.15 g QE/g of extract). Similar patterns were observed in TPC. The hexane extract contained the lowest TPC (50.953 ± 0.15 mg GAE/g of extract), whereas the methanolic extract had the highest polyphenol levels (485.42 ± 1.04 mg GAE/g of extract). In comparison to previous studies, Tagnaout et al. (2020) reported an average TPC and TFC of 19.45 ± 0.19 mg GAE/g and 2.46 ± 0.11 mg QE/g, respectively, in the aqueous extract of the plant. The same study reported that the hydroethanolic extract of *O. elongatum* contained a TPC of 44.98 ± 0.19 mg GAE/g of extract and a TFC of 1.72 ± 0.08 mg QE/g. Similarly, Yousfi et al. (2020) showed that the methanolic extract from *O. elongatum* leaves had a TPC of 153.22 ± 2.67 mg GAE/g and a TFC of 5.02 ± 0.26 mg QE/g. Interestingly, the TPC and TFC values obtained in our study are significantly higher than those reported previously. This inconsistency can be attributed to various factors, including geographic location, weather conditions, and harvest time. Moreover, the choice of the extraction method and the plant tissue used can also affect the phytochemical content and diversity (Mahdi et al., 2022).

It is well established that a higher quantity of TPC is suggested to increase the antimicrobial efficiency of medicinal plants due to their capacity to interfere with microbial growth and cellular constituents in microorganisms such as bacteria, fungi, and yeast (Aguilar-Veloz et al., 2020). Therefore, the richness of *O. elongatum* in polyphenol phytochemicals can potentially exert antimicrobial effects through multiple mechanisms, including disrupting their cell membranes, inhibiting their metabolic processes, and interfering with their DNA replication (Daglia, 2012; Aguilar-Veloz et al., 2020). In this regard, more guided studies would be required to uncover the underpinning mechanisms of the plant’s constituents.

Antioxidants derived from plants are known to play a crucial role in combating phytopathogens. In fact, plant pathogens generate reactive oxygen species (ROS) during their attack on plant tissues, leading to oxidative stress and damage (Koval et al., 2020). However, plant antioxidants neutralize these ROS and protect plant cells by scavenging free radicals and enhancing the plant’s resistance to phytopathogens (De Gara et al., 2003; Koval et al., 2020). Thus, harnessing the potential of plant antioxidants such as flavonoids and phenolic acids offers an eco-friendly approach to combat plant diseases. Therefore, the antioxidant activities of *O. elongatum* extracts were evaluated *in vitro* and showed that the DPPH radical scavenging abilities of the tested extracts demonstrated significant variations. The methanolic extract exhibited the highest scavenging capacity (EC_50_ = 87.66 ± 0.57 μg/mL), followed by the hexane and aqueous extracts, while the chloroform extract displayed the lowest antioxidant activity (EC_50_ = 162.55 ± 0.43 μg/mL). These results align with the findings reported by Tagnaout et al. (2020), who evaluated the DPPH radical scavenging potential of the residual aqueous fraction (EC_50_ = 0.856 ± 0.003 mg/mL) and the hydroethanolic extract of *O. elongatum* (EC_50_ = 0.218 ± 0.003 mg/mL), and by Oualili et al. (2019), who assessed the antioxidant effect of the plant using the ferric reducing ability of plasma (FRAP) method with EC_50_ = 1.2 mg/mL.

To the best of our knowledge, this study is the first report that investigates the inhibitory effects of *O. elongatum* extracts on *P. infestans*, the causative agent of light blight disease. In the *in vitro* experiments, both the aqueous and organic extracts demonstrated significant inhibitory effects on the mycelial growth of *P. infestans*. The effects varied depending on the extract type and concentration. The methanolic extract exhibited the most potent inhibitory effect at concentrations ranging from 1 to 5 mg/mL, followed by the chloroform extract, and then the hexane extract. Specifically, the methanolic extract at 5 mg/mL and the aqueous extract at 10 mg/mL induced the highest inhibition rate of 100%. A dose-dependent effect was observed with the aqueous extract. The antimicrobial activities of *O. elongatum* were previously tested against many species of bacteria and fungi (Amakran et al., 2014; Douhri, 2014; Boukhira et al., 2020). Notably, the vast majority of studies have been devoted to the plant’s essential oil (EO). In 2013, Fadel et al. evaluated the antifungal effects of the organic extracts of *Origanum compactum*, mainly hexane, chloroform, and methanol, against the plant pathogenic fungus *Penicillium digitatum* (Fadel et al., 2013). This study showed that the methanol extract at 80% was very effective in controlling the growth of the fungus with 100% inhibition at the concentration of 25 mg/mL. Similarly, we showed here that *O. elongatum* methanolic extract at 5 mg/mL induced a complete inhibition (100%) of *P. infestans*. The antifungal potency of the methanolic extract over chloroform and hexane extracts is most likely due to the optimal efficacy of methanol in extracting bioactive phytochemical constituents (Truong et al., 2019).

Notably, although the aqueous extracts from many *Origanum* species have been assessed against *P. infestans* (Burbano-David et al., 2021; Pinto et al., 2021), this study is the first of its kind to test the aqueous extract of *O. elongatum*. Elsewhere, the aqueous extract of *O. compactum* tested at 25 mg/mL showed a significant capacity to control the green mold fungus *P. digitatum* with an inhibition rate of 100% (Fadel et al., 2013). Additionally, the aqueous extracts of both wild and domesticated *Origanum vulgare* showed remarkable inhibitory effects against *P. infestans* with the maximum effect at 100 mg/mL (Burbano-David et al., 2021).

The positive correlation between biocontrol activity and TFC in all extracts can be explained by the fact that flavonoids are a subclass of phenolic compounds that are known to have antimicrobial properties (Baliyan et al., 2022). Elsewhere, a moderate correlation was found between DPPH antioxidant activity and rosmarinic acid, a plant polyphenol, in *O. vulgare* L. (Jafari Khorsand et al., 2022). Other studies have reported significant correlations between the antioxidant activity, TPC, TFC, and antimicrobial activity of medicinal plants against *Staphylococcus aureus* and *Escherichia coli* (Fahmideh et al., 2019). Kengne et al. (2023) investigated the phytochemical profile and antifungal and antioxidant properties of two herbs (*Tristemma mauritianum* and *Crassocephalum bougheyanum*) and found that the TPC and total TFC present in the extracts of these herbs were highly correlated with their antifungal activity.

The most active extract of *O. elongatum*, namely, the methanolic extract, was shown to contain a total of 56 components that are provisionally classified into different classes. These consist of phenolic acids, flavonoids, organic acids, tannins, and coumarins. Previously, phenolic acids such as caffeic acid, rosmarinic acid, and their derivatives were found in plants of the Lamiaceae family, including oregano. Melitric acids A and B were also identified by Chroho et al. (2022) in the hydroethanolic extract of *O. compactum* aerial parts. The identified compounds in this study were shown to possess potent bioprotectant potential against pathogens, particularly fungi. For instance, Chong et al. (2009) have investigated the antimicrobial activity and fungitoxicity of syringic acid, caffeic acid, and 4-hydroxybenzoic acid against *Ganoderma boninense*, which causes basal stem rot in oil palms. Syringic acid, which was identified in our study, was shown to exhibit the highest antifungal activity against this pathogen. Hence, the significant antifungal effect of *O. elongatum* extracts may be attributed to the abundance of syringic acid, rosmarinic acid, salvianolic acid B, and naringenin along with the potential synergistic effects induced by other compounds such as coumaric acid, salvianolic acids (A, J, and K) and melitric acids (A and B), which have been proven in numerous studies to exhibit antifungal properties (Ferreira et al., 2021; Sharma and Manhas, 2022; Sukhikh et al., 2022).

Additionally, this study is the first study to investigate the AUDPC and the effect of *O. elongatum* extracts on late blight disease severity. The results showed that the highest inhibitory effect against *P. infestans* was recorded in 25 mg/mL of the AE extract, while the lowest effect was recorded at the concentration of 10 mg/mL. Using ME, CE, and HE, 20 mg/mL demonstrated the maximum efficacy against *P. infestans*. Similar results were recorded using extracts of other plant species such as an aqueous extract from *Reynoutria sachalinensis* against *Leveillula taurica* (Powdery Mildew of Tomato) (Konstantinidou-Doltsinis et al., 2006), botanical extracts against the sheath blight disease of rice (*Rhizoctonia solani*) (Konstantinidou-Doltsinis et al., 2006), and aqueous extracts from *Bonellia flammea* and *Acalypha gaumeri* against leaf blight (caused by *Alternaria* spp.) (Vargas-Díaz et al., 2022). Ultimately, the efficacy of *O. elongatum* extracts reported in this study is more likely explained by their high contents of polyphenol and flavonoid compounds as well as organic acids, tannins, and coumarins capable of targeting microbial phytopathogens.

## 5 Conclusion

*Origanum elongatum* is a Moroccan endemic medicinal plant with a large spectrum of antimicrobial activities. In this study, we demonstrated for the first time, both *in vitro* and *in vivo*, that *O. elongatum* aqueous and organic (methanol, hexane, and chloroform) extracts are endowed with significant inhibitory effect against *P. infestans*, the causative agent of late blight in potato crops. The biocontrol efficiency on the fungus growth *in vitro* and disease severity *in vivo* was dependent on the type of extract and the concentration applied. The methanolic extract exhibited the most potent antifungal activity. LC–MS/MS profiling revealed the presence of many bioactive compounds such as organic acids, phenolic acids, flavonoids, tannins, and coumarins, which could be responsible for the biocontrol efficacy of the plant extracts. Nevertheless, further extensive investigations are necessary to explore the plant’s ability to combat other species of plant pathogens through its antimicrobial properties. Additionally, it is crucial to conduct fractionation and guided bioassays to identify the most active phytoconstituents in the plant and elucidate the involved mechanisms of action. Finally, by harnessing the inherent antimicrobial and antioxidant properties of the plant compounds, bioformulation experiments could offer a promising avenue for eco-friendly alternatives to conventional fungicides.

## Data availability statement

The original contributions presented in the study are included in the article/supplementary material. Further inquiries can be directed to the corresponding authors.

## Author contributions

AH: Conceptualization, Data curation, Formal analysis, Methodology, Software, Writing – original draft, Writing – review & editing. GE: Formal analysis, Investigation, Software, Supervision, Writing – review & editing. F-AD: Data curation, Formal analysis, Software, Writing – original draft. NT: Formal analysis, Resources, Software, Writing – review & editing. NS: Formal analysis, Software, Writing – review & editing. MS: Formal analysis, Resources, Software, Writing – review & editing. SE: Formal analysis, Software, Writing – review & editing. S-EL: Formal analysis, Software, Writing – review & editing. MB: Formal analysis, Supervision, Writing – review & editing. RL: Conceptualization, Funding acquisition, Investigation, Methodology, Project administration, Resources, Supervision, Validation, Writing – review & editing.

## References

[B1] Abdelaali B. El Menyiy N. El Omari N. Benali T. Guaouguaou F.-E. Salhi N.. (2021). Phytochemistry, toxicology, and pharmacological properties of *origanum elongatum* . Evidence-Based Complementary Altern. Med. 2021, e6658593. doi: 10.1155/2021/6658593 PMC8225437 34221086

[B2] Agrios G. N. (2005). Plant pathology. 5th Edition (Amsterdam: Elsevier Academic Press).

[B3] Aguilar-Veloz L. M. Calderón-Santoyo M. Vazquez Gonzalez Y. Ragazzo-Sánchez J. A. (2020). Application of essential oils and polyphenols as natural antimicrobial agents in postharvest treatments: Advances and challenges. Food Sci. Nutr. 8, 2555–2568. doi: 10.1002/fsn3.1437 32566173 PMC7300048

[B4] Amakran A. Hamoudane M. Ramdan B. Lamarti A. Pagniez F. le Pape P.. (2014). Antifungal activity of the essential oil of *origanum elongatum* on candida, aspergillus and rhizopus species. J. Mycologie Medicale 2, e78. doi: 10.1016/j.mycmed.2014.01.078

[B5] Bajpai V. K. Lee T. J. Kang S. C. (2009). Chemical composition and in *vitro* control of agricultural plant pathogens by the essential oil and various extracts of *Nandina domestica Thunb* . J. Sci. Food Agric. 89, 109–116. doi: 10.1002/jsfa.3416

[B6] Bakha M. El Mtili N. Machon N. Aboukhalid K. Amchra F. Z. Khiraoui A.. (2020). Intraspecific chemical variability of the essential oils of Moroccan endemic *Origanum elongatum L.* (Lamiaceae) from its whole natural habitats. Arabian J. Chem. 13, 3070–3081. doi: 10.1016/j.arabjc.2018.08.015

[B7] Baliyan S. Mukherjee R. Priyadarshini A. Vibhuti A. Gupta A. Pandey R. P.. (2022). Determination of antioxidants by DPPH radical scavenging activity and quantitative phytochemical analysis of *ficus religiosa* . Molecules 27, 1326. doi: 10.3390/molecules27041326 35209118 PMC8878429

[B8] Belay D. W. Asfaw Z. Lulekal E. Kassa B. (2021). Farmers’ management of potato (*Solanum tuberosum L.*) late blight (*Phytophtora infestans* (Mont.) de Bary) and sprouting in Shashemene and West Shewa districts, Ethiopia. Cogent Food Agric. 7, 1925432. doi: 10.1080/23311932.2021.1925432

[B9] Bitchagno G. T. M. El Bouhssini M. Mahdi I. Ward J. L. Sobeh M. (2022). Toward the allelopathy of *peganum* sp. and related chemical constituents in agriculture. Front. Plant Sci. 12. doi: 10.3389/fpls.2021.796103 PMC8813868 35126420

[B10] Boukhira S. Bousta F. Moularat S. Abdellaoui A. Ouaritini Z. B. Bousta D. (2020). Evaluation of the preservative properties of *origanum elongatum* essential oil in a topically applied formulation under a challenge test. Phytothérapie 18, 92–98. doi: 10.3166/phyto-2018-0067

[B11] Burbano-David D. Lagos-Mora L. E. Álvarez-Ordoñez S. Chañag-Miramag H. A. (2021). Sensitivity of *Phytophthora infestans* to aqueous extracts of *Lippia origanoides* and *Origanum vulgare* . Agronomía Mesoamericana 32, 149–162. doi: 10.15517/am.v32i1.40573

[B12] Chen Y. F. Chen W. Huang X. Hu X. Zhao J. T. Gong Q.. (2013). Fusarium wilt-resistant lines of Brazil banana (Musa spp., AAA) obtained by EMS-induced mutation in a micro-cross-section cultural system. Plant Pathol. 62, 112–119. doi: 10.1111/j.1365-3059.2012.02620.x

[B13] Chong K. P. Rossall S. Atong M. (2009). *In vitro* antimicrobial activity and fungitoxicity of syringic acid, caffeic acid and 4-hydroxybenzoic acid against ganoderma boninense. JAS 1, p15. doi: 10.5539/jas.v1n2p15

[B14] Chroho M. Bouymajane A. Aazza M. Oulad El Majdoub Y. Cacciola F. Mondello L.. (2022). Determination of the phenolic profile, and evaluation of biological activities of hydroethanolic extract from aerial parts of *origanum compactum* from Morocco. Molecules 27, 5189. doi: 10.3390/molecules27165189 36014429 PMC9413242

[B15] Daglia M. (2012). Polyphenols as antimicrobial agents. Curr. Opin. Biotechnol. 23, 174–181. 21925860 10.1016/j.copbio.2011.08.007

[B16] De Gara L. de Pinto M. C. Tommasi F. (2003). The antioxidant systems vis-à-vis reactive oxygen species during plant–pathogen interaction. Plant Physiol. Biochem. 41, 863–870. doi: 10.1016/S0981-9428(03)00135-9

[B17] Dewitte K. Landschoot S. Carrette J. Audenaert K. Haesaert G. (2019). Exploration of essential oils as alternatives to conventional fungicides in lupin cultivation. Org. Agr. 9, 107–116. doi: 10.1007/s13165-018-0212-3

[B18] Douhri B. (2014). Hepatoprotective Effect of *Origanum elongatum* against Carbon Tetrachloride (CCl4) Induced Toxicity in Rats. EJMP 4, 14–28. doi: 10.9734/EJMP/2014/5132

[B19] El Attari H. Chefira K. Rchid H. Nmila R. (2019). Corrosion inhibition study of the *Origanum elongatum* extract: electrochemical, gravimetric and adsorption isotherms studies in 0.5 m sulfuric acid. Res. J. Pharmaceutical Biol. Chem. Sci. 10, 1491–1507.

[B20] El Harsal A. Ibn Mansour A. Skali Senhaji N. Ouardy Khay E. Bouhdid S. Amajoud N.. (2018). Influence of extraction time on the yield, chemical composition, and antibacterial activity of the essential oil from *Origanum elongatum* (E. & M.) Harvested at Northern Morocco. J. Essential Oil Bearing Plants 21, 1460–1474. doi: 10.1080/0972060X.2019.1572545

[B21] El Khetabi A. Lahlali R. Askarne L. Ezrari S. El Ghadaroui L. Tahiri A.. (2020). Efficacy assessment of pomegranate peel aqueous extract for brown rot (Monilinia spp.) disease control. Physiol. Mol. Plant Pathol. 110, 101482. doi: 10.1016/j.pmpp.2020.101482

[B22] Fadel F. Ben Hmamou D. Salghi R. Chebli B. Benali O. Zarrouk A.. (2013). Antifungal activity and anti-corrosion inhibition of *Origanum compactum* extracts. Int. J. Electrochem Sci. 8, 11019–11032. doi: 10.1016/S1452-3981(23)13166-X

[B23] Fahmideh L. Mazaraie A. Tavakoli M. (2019). Total phenol/flavonoid content, antibacterial and DPPH free radical scavenging activities of medicinal plants. J. Agric. Sci. Technol. 21, 1459–1471.

[B24] Ferreira P. S. Victorelli F. D. Rodero C. F. Fortunato G. C. Araújo V. H. S. Fonseca-Santos B.. (2021). p-Coumaric acid loaded into liquid crystalline systems as a novel strategy to the treatment of vulvovaginal candidiasis. Int. J. Pharmaceutics 603, 120658. doi: 10.1016/j.ijpharm.2021.120658 33964336

[B25] Figueredo A. J. Vásquez G. Brumbach B. H. Schneider S. M. R. Sefcek J. A. Tal I. R.. (2006). Consilience and Life History Theory: From genes to brain to reproductive strategy. Dev. Rev. 26, 243–275. doi: 10.1016/j.dr.2006.02.002

[B26] Guenthner J. Michael K. Nolte P. (2001). Potato late blight’s impact on growers. Potato Res. 44, 121–125. doi: 10.1007/BF02410098

[B27] Haesaert G. Vossen J. H. Custers R. De Loose M. Haverkort A. Heremans B.. (2015). Transformation of the potato variety Desiree with single or multiple resistance genes increases resistance to late blight under field conditions. Crop Prot. 77, 163–175. doi: 10.1016/j.cropro.2015.07.018

[B28] Haque E. Irfan S. Kamil M. Sheikh S. Hasan A. Ahmad A.. (2016). Terpenoids with antifungal activity trigger mitochondrial dysfunction in *Saccharomyces cerevisiae* . Microbiology 85, 436–443. doi: 10.1134/S0026261716040093 28853775

[B29] Hmidani A. Bouhlali E. dine T. Ajebli M. Khouya T. Benlyas M.. (2021). *In vitro* investigation of antioxidant and antihemolytic activities of three Lamiaceae species from Morocco. Beni-Suef Univ. J. Basic Appl. Sci. 10, 27. doi: 10.1186/s43088-021-00116-9

[B30] Jafari Khorsand G. Morshedloo M. R. Mumivand H. Emami Bistgani Z. Maggi F. Khademi A. (2022). Natural diversity in phenolic components and antioxidant properties of oregano (*Origanum vulgare L.*) accessions, grown under the same conditions. Sci. Rep. 12, 5813. doi: 10.1038/s41598-022-09742-4 35388099 PMC8987097

[B31] Kengne I. C. Fankam A. G. Yamako E. K. Tamokou J.-D.-D. (2023). Phytochemical Analysis, Antifungal, and Antioxidant Properties of Two Herbs (*Tristemma mauritianum* and *Crassocephalum bougheyanum*) and One Tree (*Lavigeria macrocarpa*) Species. Adv. Pharmacol. Pharm. Sci. 2023, e2565857. doi: 10.1155/2023/2565857 PMC9891821 36742131

[B32] Kisiriko M. Anastasiadi M. Terry L. A. Yasri A. Beale M. H. Ward J. L. (2021). Phenolics from medicinal and aromatic plants: characterisation and potential as biostimulants and bioprotectants. Molecules 26, 6343. doi: 10.3390/molecules26216343 34770752 PMC8588183

[B33] Konstantinidou-Doltsinis S. Markellou E. Kasselaki A.-M. Fanouraki M. N. Koumaki C. M. Schmitt A.. (2006). Efficacy of Milsana®, a Formulated Plant Extract from Reynoutria sachalinensis, against Powdery Mildew of Tomato (Leveillula taurica). Biocontrol 51, 375–392. doi: 10.1007/s10526-005-5247-1

[B34] Koval D. Plocková M. Kyselka J. Skřivan P. Sluková M. Horáčková Š. (2020). Buckwheat secondary metabolites: potential antifungal agents. J. Agric. Food Chem. 68, 11631–11643. doi: 10.1021/acs.jafc.0c04538 32985180

[B35] Lamichhane J. R. Dachbrodt-Saaydeh S. Kudsk P. Messéan A. (2016). Toward a reduced reliance on conventional pesticides in European agriculture. Plant Dis. 100, 10–24. doi: 10.1094/PDIS-05-15-0574-FE 30688570

[B36] Lamichhane J. R. Osdaghi E. Behlau F. Köhl J. Jones J. B. Aubertot J.-N. (2018). Thirteen decades of antimicrobial copper compounds applied in agriculture. A review. Agron. Sustain. Dev. 38, 28. doi: 10.1007/s13593-018-0503-9

[B37] Mahdi I. Bakrim W. B. Bitchagno G. T. M. Annaz H. Mahmoud M. F. Sobeh M. (2022). Unraveling the phytochemistry, traditional uses, and biological and pharmacological activities of *thymus algeriensis* boiss. & Reut. Oxid. Med. Cell. Longevity 2022, e6487430. doi: 10.1155/2022/6487430 PMC9159826 35663202

[B38] Mazumdar P. Singh P. Kethiravan D. Ramathani I. Ramakrishnan N. (2021). Late blight in tomato: insights into the pathogenesis of the aggressive pathogen *Phytophthora infestans* and future research priorities. Planta 253, 119. doi: 10.1007/s00425-021-03636-x 33963935

[B39] Moura A. B. Backhouse D. de Souza Júnior I. T. Gomes C. B. (2022). “Soilborne pathogens,” in Subsoil constraints for crop production. Eds. de Oliveira T. S. Bell R. W. (Cham: Springer International Publishing), 199–224. doi: 10.1007/978-3-031-00317-2_9

[B40] Moussaoui N. (2013). Antibacterial and antiviral activities of essential oils of Northern Moroccan plants. BBJ 3, 318–331. doi: 10.9734/BBJ/2013/3596

[B41] Najdabbasi N. Mirmajlessi S. M. Dewitte K. Landschoot S. Mänd M. Audenaert K.. (2020). Biocidal activity of plant-derived compounds against *Phytophthora infestans*: An alternative approach to late blight management. Crop Prot. 138, 105315. doi: 10.1016/j.cropro.2020.105315

[B42] Oualili H. Nmila R. Chibi M. Mricha A. Rchid H. Rchid H. (2019). Chemical composition and antioxidant activity of *origanum elongatum* essential oil. Pharmacognosy Res. 11, 283–289. doi: 10.4103/pr.pr_157_18

[B43] Parveen S. Wani A. H. Ganie A. A. Pala S. A. Mir R. A. (2014). Antifungal activity of some plant extracts on some pathogenic fungi. Arch. Phytopathol. Plant Prot. 47, 279–284. doi: 10.1080/03235408.2013.808857

[B44] Pinto T. Aires A. Cosme F. Bacelar E. Morais M. C. Oliveira I.. (2021). Bioactive (Poly)phenols, volatile compounds from vegetables, medicinal and aromatic plants. Foods 10, 106. doi: 10.3390/foods10010106 33419090 PMC7825428

[B45] Qin C.-F. He M.-H. Chen F.-P. Zhu W. Yang L.-N. Wu E.-J.. (2016). Comparative analyses of fungicide sensitivity and SSR marker variations indicate a low risk of developing azoxystrobin resistance in *Phytophthora infestans* . Sci. Rep. 6, 20483. doi: 10.1038/srep20483 26853908 PMC4745062

[B46] Ramzi H. Ismaili M. R. Aberchane M. Zaanoun S. (2017). Chemical characterization and acaricidal activity of *Thymus satureioides* C. & B. and *Origanum elongatum* E. & M. (Lamiaceae) essential oils against *Varroa destructor* Anderson & Trueman (Acari: Varroidae). Ind. Crops Products 108, 201–207. doi: 10.1016/j.indcrop.2017.06.031

[B47] Saldaña G. Cebrián G. Abenoza M. Sánchez-Gimeno C. Álvarez I. Raso J. (2017). Assessing the efficacy of PEF treatments for improving polyphenol extraction during red wine vinifications. Innovative Food Sci. Emerging Technol. 39, 179–187. doi: 10.1016/j.ifset.2016.12.008 PMC5312662 28239292

[B48] Scott P. Bader M. K.-F. Burgess T. Hardy G. Williams N. (2019). Global biogeography and invasion risk of the plant pathogen genus Phytophthora. Environ. Sci. Policy 101, 175–182. doi: 10.1016/j.envsci.2019.08.020

[B49] Sharma M. Manhas R. K. (2022). Biocontrol potential of *Streptomyces* sp. M4 and salvianolic acid B produced by it against Alternaria black leaf spot. Microbial Pathogenesis 173, 105869. doi: 10.1016/j.micpath.2022.105869 36356795

[B50] Soylu E. M. Soylu S. Kurt S. (2006). Antimicrobial activities of the essential oils of various plants against tomato late blight disease agent. Phytophthora infestans. Mycopathologia 161, 119–128. doi: 10.1007/s11046-005-0206-z 16463095

[B51] Sukhikh S. Ivanova S. Babich O. Larina V. Krol O. Prosekov A.. (2022). Antimicrobial screening and fungicidal properties of *Eucalýptus globulus* ultrasonic extracts. Plants 11, 1441. doi: 10.3390/plants11111441 35684214 PMC9182981

[B52] Tagnaout I. Zerkani H. Amine S. Fadili K. Benhlima N. Bouzoubaa A.. (2020). Phenolic composition and antioxidant potential of different solvent extracts of the endemic *Origanum elongatum* (Bonnet) Emberger& Maire. Mediterr. J. Chem. 10, 146–154. doi: 10.13171/mjc10202002111248ittz

[B53] Truong D.-H. Nguyen D. H. Ta N. T. A. Bui A. V. Do T. H. Nguyen H. C. (2019). Evaluation of the use of different solvents for phytochemical constituents, antioxidants, and *in vitro* anti-inflammatory activities of *severinia buxifolia* . J. Food Qual. 2019, e8178294. doi: 10.1155/2019/8178294

[B54] Vargas-Díaz A. A. Cristóbal-Alejo J. Canto-Canché B. Gamboa-Angulo M. M. Vargas-Díaz A. A. Cristóbal-Alejo J.. (2022). Aqueous extracts from *Acalypha gaumeri* and *Bonellia flammea* for leaf blight control in chrysanthemum (*Chrysanthemum morifolium*). Rev. mexicana fitopatología 40, 40–58. doi: 10.18781/r.mex.fit.2109-1

[B55] Wang T. Gao C. Cheng Y. Li Z. Chen J. Guo L.. (2020). Molecular diagnostics and detection of oomycetes on fiber crops. Plants 9, 769. doi: 10.3390/plants9060769 32575466 PMC7355704

[B56] Wang X. Zheng K. Cheng W. Li J. Liang X. Shen J.. (2021). Field application of star polymer-delivered chitosan to amplify plant defense against potato late blight. Chem. Eng. J. 417, 129327. doi: 10.1016/j.cej.2021.129327

[B57] Wightwick A. Walters R. Allinson G. Reichman S. Menzies N. (2010). Environmental risks of fungicides used in horticultural production systems. Fungicides 1, 273–304.

[B58] Xu W. Zhang F. Luo Y. Ma L. Kou X. Huang K. (2009). Antioxidant activity of a water-soluble polysaccharide purified from *Pteridium aquilinum* . Carbohydr. Res. 344, 217–222. doi: 10.1016/j.carres.2008.10.021 19036355

[B59] Yousfi K. E. Greche H. Misbahi H. Cheikh R. B. (2020). Phytochemical evaluation and vasodilatory activity of *O. elongatum*, C. salviifolius and *C. laurifolius* . Moroccan J. Chem. 8, 8–234. doi: 10.48317/IMIST.PRSM/morjchem-v8i1.19097

[B60] Yuen J. (2021). Pathogens which threaten food security: *Phytophthora infestans*, the potato late blight pathogen. Food Sec. 13, 247–253. doi: 10.1007/s12571-021-01141-3

